# Impact of Metabolic Syndrome Longitudinal Changes on Cognitive Health: A 10‐Year Follow‐up Study in Middle‐Aged Adults

**DOI:** 10.1002/alz.087861

**Published:** 2025-01-09

**Authors:** Hsiu‐Fen Lin, Cheng‐Sheng Chen

**Affiliations:** ^1^ Kaohsiung Medical University, Kaoshiung Taiwan; ^2^ Kaohsiung Medical University Hospital, Kaohsiung Medical University, Kaohsiung, NA Taiwan

## Abstract

**Background:**

Metabolic syndrome (MS) has been linked to cognitive functions, yet limited research has explored the longitudinal effects of changes in MS on cognitive performance. This study aims to investigate the predictive impact of longitudinal MS status on cognitive performance and dissect its sex‐specific influence over a 10‐year follow‐up.

**Method:**

In this community‐based prospective cohort study, 766 healthy subjects underwent baseline MS status assessment between 2006 and 2011. Changes in MS status and cognitive performance were evaluated from 2016 to 2021 using the Montreal Cognitive Assessment (MoCA). MS change status was categorized into four groups: never MS, ever MS with improvement, newly onset MS, and persistent MS. Linear regression analysis was used to assess the impact of longitudinal MS changes on endpoint cognition status while controlling for covariates. Categorical variables were dummy‐coded, and sex‐specific effects were examined by reanalyzing the data divided by sex.

**Result:**

Subjects had a mean age of 54.50 ± 8.03 years at baseline, with a mean follow‐up duration of 10.40 ± 0.79 years; 39.2% were men. The MoCA score in the persistent MS group was significantly lower than that in the never MS group (adjusted p = 0.020). However, no significant differences in MoCA scores were observed between the never MS group and the ever MS with improvement group (adjusted p = 0.759) or the newly onset MS group (adjusted p = 0.469). Further analysis of cognitive domains revealed that memory and language were the most significantly affected. In assessing sex‐specific effects, a significant association was found only in women, with no significant associations observed in men.

**Conclusion:**

Persistent MS status contributes to cognitive decline over a 10‐year period, contrasting with the lack of impact in those with improving or newly onset MS status. This effect is particularly pronounced in middle‐aged women. The findings suggest that modifying MS status among middle‐aged individuals, especially women, may be beneficial in preventing future cognitive decline.

